# Perceptions of Healthcare Workers (HCWs) towards childhood immunization and immunization services in Fiji: a qualitative study

**DOI:** 10.1186/s12887-022-03665-9

**Published:** 2022-10-21

**Authors:** Preeti Balgovind, Masoud Mohammadnezhad

**Affiliations:** 1 Ministry of Health and Medical Services, Suva, Fiji Islands., National University, Suva, Fiji Islands; 2 School of Nursing and Healthcare Leadership, University of Bradford, Bradford, United Kingdom

**Keywords:** Healthcare workers, Childhood immunization, Perceptions, Fiji Immunisation schedule

## Abstract

**Introduction:**

Childhood immunization has been globally recognized as the single most effective strategy in preventing childhood diseases and mortality. The perceptions of healthcare workers are important as their behavior and attitudes influence parental decision–making process. This research aimed to explore the factors that influence healthcare workers’ experience and perceptions about delivering childhood immunization in Fiji.

**Materials and methods:**

A qualitative study was conducted in three randomly selected health centers in Suva, Fiji from March 1st to April 5th, 2021. Five focus group discussions were conducted with healthcare workers who were chosen purposively, had worked in the health center for at least 6 months and included either gender. Those that did not consent or did not meet the inclusion criteria were excluded. The interviews were guided by semi–structured open–ended questionnaire and were recorded into a digital voice recorder. The data were coded, sorted, and then categorized into themes, and transcribed onto Microsoft Word. Thematic analysis was utilized to sort the key phrases from the recorded interviews.

**Results:**

There were a total of 22 participants for the focus group discussions, with their ages ranging from 25 to 51 years, included 3 medical officers, 1 nurse practitioner and 18 registered nurses. Three major themes emerged, which included: healthcare worker factors, parental factors and health system factors. Subthemes identified from the healthcare worker factors were worker knowledge and attitudes. The subtheme for parental factors that emerged were defaulters, parental attitudes, perceived behavior and religious beliefs. For health system factors the subthemes were service delivery, registration, infrastructure, staff turnover, staff training and changes to the immunization schedule.

**Conclusion:**

Some of the perceived barriers reported by the healthcare workers were parental religious beliefs, parental knowledge and attitude, social or physical factors (finances, transportation, childcare and work conflicts), access to health services, immunization services and policies, hours of operation, waiting time and missed opportunities. Health workers acknowledged that they have an important role to play in immunization as they are the source of information and motivation for parents. Further studies are needed to be conducted nationally to determine the perceptions of healthcare workers towards immunization and how the services can be improved on a national level.

**Supplementary Information:**

The online version contains supplementary material available at 10.1186/s12887-022-03665-9.

## Background

Childhood immunization against common childhood diseases is one important strategy to keep children healthy [1–5]. It has been the most cost–effective public health intervention, saving an estimated 2–3 million lives globally each year and is critical for reducing the global child morbidity and mortality [1, 6, 7]. A study done from 1994 to 2003 in the United States of America, found that routine childhood immunization has prevented 322 million cases of illnesses and 732,000 premature deaths from Vaccine Preventable Diseases (VPDs), resulting in a net savings of an estimated $295 billion in direct medical costs [8, 9].

Immunization coverage is an important indicator to track and guide immunization programs at the global, national, and subnational level [10]. A study on the Fiji National Immunization Coverage Survey in 2013 found that 91% of children nationally had received all the required 10 doses of the immunization schedule and were fully immunized with data ranging from 87% in the Eastern Division to 94% in Northern Division [11]. According to WHO, global coverage has dropped from 86% to 2019 to 83% in 2020, an estimated 23 million children < 1 years of age not receiving basic vaccines in 2020, the highest number since 2009 and the number of completely unimmunized children has increased by 3. 4 million [12].

This could be due to vaccine delays or refusals, a lack of trust in the importance, safety, or effectiveness of vaccines, alongside persisting access issues [13]. According to the World Health Organization (WHO), during 2019 only 86% of children received their third dose of the Diphtheria–Tetanus–Pertussis (DTP_3_) vaccines, 20 million children missed out on measles, diphtheria, and tetanus vaccines and many vaccination campaigns were cancelled [12]. This slowdown could continue as countries focus on efforts to control the COVID–19 pandemic and the introduction of the COVID–19 vaccines [14, 15]. Many resources and personnel have been diverted to support the COVID–19 response, significant disruptions to immunization service provision have occurred, clinics have been closed or hours reduced, reluctance to seek healthcare because of fear of transmission or challenges experienced reaching services due to lockdown measures and transportation disruptions [14].

Many children do not receive the recommended vaccines as their parents or caregivers do not understand why immunization is essential, do not understand how, where or when to get their children immunized, cannot access health facilities or have concerns or doubts about the vaccine safety and efficacy [16]. High dropout between early and final doses of the primary vaccine series may indicate health system barriers to reattendance, failure to educate mothers of the need to return, or inadequate tracking of children registered at the health facility [17]. Healthcare Workers (HCWs) are important sources of information regarding immunization for parents [18, 19] and their recommendations are important drivers of vaccine uptake. Midwives can also be an important source of information to parents about the recommended vaccines and immunization schedule for their children [20].

Some parents source immunization information, through the internet, friends, family, television, radio, and newspapers [21–23]. Missed immunization does not necessarily mean that parents have made a conscious decision not to immunize, but can be due to forgetting the allotted date, lack of time, illness in the family or having other childcare commitments [24]. The roles of HCWs are to improve access to health care and health–seeking behavior [25] and whether involved in immunization services at their facility or not, should know the full schedule and the purpose of each vaccine.

HCWs involved in immunization in Fiji are the staff nurses based at the Maternal Child Health Clinics (MCHCs) of the health centers around Fiji. Fiji Immunization Schedule has a comprehensive schedule, and it starts from birth right up to 18 months of age includes tuberculosis (TB), poliomyelitis, measles, DTP, Hepatitis B, mumps, rubella, pneumococcal, rotavirus and *Haemophilus influenzae* type b (Hib) vaccines [26]. Fiji Maternal Child Health (MCH) cards have information regarding the birth details, immunization schedule, weight and growth chart, the next clinic date, developmental milestones chart and information on danger signs [26]. The clinics are done up to the age of 5 years of age. Completed childhood immunization schedule is mandatory for entry into the schooling system in Fiji.

There is an absence of research in Fiji on HCWs’ perceptions towards childhood immunization, therefore, this research aims to identify the difficulties, the perceived barriers and explore HCWs attitudes and ways to address them. The research could assist in the improvement of the immunization services and delivery. No study could be found that explored the perceptions of HCWs towards immunization in Fiji. Therefore, this study aims to bridge this gap between the findings from the western countries and the local context.

## Methods

### Study design and setting

This was a qualitative study using focus group discussions conducted with HCWs’ to explore their perceptions on childhood immunization and immunization services conducted in Suva, Fiji from March 1st, 2021, to April 5th, 2021. Qualitative study design allows to explore subjective issues like beliefs, values, emotions, feelings, motivations, attitudes, perceptions, and barriers that may explain why certain behavior towards immunization exists [27, 28]. The research was carried out in the MCHCs of three purposively selected health centers and were chosen as they gave a mixture of peri–rural, peri–urban and urban health facilities.

### Study sample

The study population was the HCWs working at the three health centers and those that met the inclusion and exclusion criteria. The inclusion criteria were medical officers, staff nurses that worked in the MCHCs, zone nursing department and the General Outpatient Department (GOPD) and had a minimum of 6 months of working experience, of either gender, held valid Fiji practicing license and of any ethnicity. GOPD staff do not administer the vaccines but are required know the Fiji Immunization Schedule. MCHC nurses and the Zone nurses work in the same department, and they are the ones that administer the vaccines, and follow up on their clients.

The exclusion criteria were those that did not consent to participate in the study and those not meeting the inclusion criteria. Purposive sampling was used to choose the participants for the study, which is the deliberate choosing of a participant due to the qualities a participant may possess which, in this study included knowledge of the Fiji Immunization Schedule, having worked at the chosen facility for at least 6 months and had adequate working experience and knowledge [29]. There were 22 participants included in this study. There were 5 focus group discussions held with each group having 4–5 HCWs.

### Data collection tools

The focus group discussions were guided by a semi–structured questionnaire (Additional File No. 1) that used 8 content–validated open–ended and probing questions, and focused on the understanding, perceptions, barriers, and communication skills of HCWs towards immunization. A demographic form was also filled by the participants to collect socioeconomic information on their age, gender, ethnicity, and years of service. The focus group discussions were conducted in English language. The questionnaire contents were validated by three experts at Fiji National University (FNU) and given to three health workers (not part of the study) to check for validity and if the questions were understandable. The questionnaire went through a peer review period where a limited number of interviews were conducted among four participants meeting the criteria to review the questions and test the questionnaire. An evaluation was obtained to assess if the questions needed any amendments.

### Study procedure

The staff at the MCHC were given flyers containing information about the research at each of the three health centers for at least 3 weeks and given an introduction of the study. Along with the introduction, an information sheet in the English language was given to all the HCWs who met the inclusion criteria. Written informed consent (with freedom to withdraw from the study at any stage) was taken from the participants and was confidentiality assured. The focus group discussions were recorded onto a voice recorder and conducted by the main researcher in a secluded area maintaining privacy and background noise prevention, lasting for at least 60 min. All participants were asked the same questions, with follow up probing question maintaining consistency all throughout.

### Data management and analysis

The points collected during the discussions, together with the recorded data were transcribed using handwritten text and then transcribed on Microsoft Word®. The audio recordings were heard two to three times to ensure that the transcribed data was exactly as in the audio. From the transcribed data, and all the unnecessary or irrelevant data was excluded. The aggregated data was compiled and presented as the results of this study. The data was then coded and entered into Microsoft Excel® and EpiInfor™ was used to calculate the percentages of participants across the different characteristics. The relevant data was analyzed using the thematic analysis to summarize all the barriers, attitudes and perceptions of the HCWs under themes. Castleberry and Nolen, (2018) has defined thematic analysis as the data analysis strategy that is commonly used approach across all qualitative designs that reduces the data into workable themes and emerging conclusions [27]. As outlined by Braun and Clarke, (2006) the thematic analysis was guided by getting familiar with the data, generating the codes, looking for themes, reviewing the themes, defining the themes and the final write up [30].

### Study rigor

For a study process to be valid, Lincoln and Guba (1986), proposed that any research should satisfy the four–dimensional criteria. The four–dimensional criteria (credibility, dependability, confirmability and transferability) were implemented to assess the quality of this research [31, 32]. The methods utilized to maintain study rigor included: ability of the study to maintain reflexibility (being aware of biases, having accountability, research being trustworthy and having clarity), use of the participants’ actual wordings in the final report, adequate time spent with each participant, range of experiences which were reported by the participants captured in final report, ability to utilize the study methods in other geographical locations, peer review of the questions and the final report, able to specify the purpose of the study, provision of a detailed description of the research methods (selection of the participants, data collection and the period of data collection) and taking notes of personal feelings and insights of the participants immediately after the focus group discussions [31, 32].

### Ethical considerations

Ethical approval was first sought from the College Health Research and Ethics Committee (CHREC) of the Fiji National University (FNU). Once obtained, approval was then gained from the Sub–Divisional Medical Officer and Medical–Officers–In–Charge of the three health centers. The HCWs were explained on how the data and their responses will be utilized. Written informed consent (with freedom to withdraw from the study at any stage) was taken from the participants and was confidentiality assured. All the data collected were stored securely on the laptop during the analysis stage and were destroyed once no longer required. Back up data was stored on the Microsoft OneDrive® and will be deleted once no longer required.

## Results

### General characteristics of the participants

A total of five focus group discussions was held with a mixture of participants in the group with medical officers, nurse practitioner and the staff nurses included in the group. There were 21 female participants and 1 male participant. Their ages ranged from less than 30 years to 51 years, 12 of which were Fijians of Indian Descent and 10 were iTaukei (Indigenous Fijians). Their years of service ranged from less than 5 years of service to more than 20 years of service. The characteristics are summarized in Table 1.

**Table 1:** Characteristics of study participants (n = 22)

Variables	n (%)
**Age**	
≤ 30 years	8 (36)
31–40 years	11 (50)
41–50 years	2 (9)
≥ 50 years	1 (5)
**Gender**	
Female	21 (95)
Male	1 (5)
**Ethnicity**	
Fijian of Indian Descent	12 (55)
iTaukei	10 (45)
**Designation**	
Medical Officer	4 (18)
Staff nurse	18 (82)
**Years of service**	
≤ 5 years	4 (18)
6–10 years	7 (32)
11–15 years	7 (32)
≥ 16 years	4 (18)
**Training in EPI**	
Yes	15 (68)
No	7 (32)

EPI = Expanded Program for Immunization

### Themes and subthemes

The responses from the participants were captured under three major themes: HCW factors, parental factors and health system factors. The themes and the subthemes are shown in the Table 2.

**Table 2:** Identified themes and subthemes of FGD

Theme	Subthemes
**1. HCW factors**	Knowledge
	Attitudes
**2. Parental factors**	Perceived parental attitude
	Cultural beliefs and practices
	Defaulters
**3. Health system factors**	Service delivery
	Registration
	Infrastructure
	Staff turnover
	Staff training
	Changes to the immunization schedule

FGD = Focus Group Discussion

### Theme 1

There were 2 subthemes found under Theme 1 – HCW knowledge and attitudes.

#### HCW knowledge

All the HCWs (n = 22) felt that they had adequate knowledge on childhood immunization and the Fiji Immunization Schedule. Most of them had undergone some studies on the schedule in their medical training. The MCHC nurses (n = 4) were the ones that gave the vaccines to the children in their clinics. Fifteen (n = 15) staff had undergone the Expanded Program for Immunization (EPI) training and seven (n = 7) had not.

“*Even though, I have not gone through the EPI training, I have been working in MCHC for more than 5 years – previously in Lautoka and for 2 months at Makoi Health Center.*” (29–year–old, MCHC staff nurse)

#### HCW attitudes

All the HCWs in the research responded that it was important for them to be advocates for immunization hence, they have positive attitudes towards immunization.

“*I have children, and they are immunized and up–to–date with their immunization. I made the effort to get them immunized as I know how important it is for them.*” (41–year–old, IMCI staff nurse)

### Theme 2

There were 3 subthemes found under Theme 2: perceived parental attitudes, cultural beliefs and practices, and defaulters.

#### Perceived parental attitudes

HCWs reported that they perceived parental attitudes as a barrier to obtaining optimal immunization coverage. They reported encountering some parents/caregivers who felt that immunization was not necessary and did not return after the initial visit. They also reported that some parents were neglectful in keeping the cards safely and that some parents are least bothered about coming at the scheduled time. Some of the staff reported that fever, pain, swelling and crying post–immunization is common.

“*One bad reaction in a relative’s child or someone they know, or even in their child, it is enough to cause distrust for the vaccines. But they don’t realize not every case is the same.*” (31–year–old, MCHC staff nurse)

#### Cultural beliefs and practices

The respondents all responded that they perceive religion as one of the factors that may be one of the decision–making factors for parents. They said that they have encountered a few parents who have not completed the immunization for their child after the initial immunization, quoting religious beliefs.

“*I have encountered two sets of parents. They said that they prayed about it and didn’t want their child to have any further immunizations.*” (29–year–old, MCHC staff nurse)

#### Defaulters

One of the staff reported that she had encountered parents who think that vaccines are harmful and would not immunize their child and prefer herbal or traditional medicine.

“*I have met few parents who have refused immunization because they think it is bad and they want to use herbal or traditional medicine. They told me they may reconsider if the child gets sick.*” (41–year–old, Zone staff nurse)

All the immunizations given in a clinic day are entered into a register for the areas that the child lives in and there is a register for outside area coverage. The outside area register is utilized for those parents that bring their child in for a scheduled immunization but do not live in that area. The respondents reported that it is hard at times to do defaulter tracing, since in Fiji it is mostly done through paper. They also reported that at times the registered addresses are not correct, or not given the updated address and contact details.

“*It is hard to trace defaulters. Because when we go to the registered address, they are not living there anymore….*” (34–year–old, Zone staff nurse)

Medical officers reported that when sick children presented to the GOPD and when their Maternal Child Health cards are checked, it is noted that they have missed doses of vaccines. The respondents reported that the children present in a serious and critical state like severe pneumonia, sepsis, septic skin lesions and severe dehydration due to severe diarrhea.

“*I have encountered very sick children coming into emergency (malnourished or in shock) or having severe respiratory diseases, and when we check the MCH card, they have missed their immunization. The immunizations are especially important, and the parents should take advantage of it as it is also given free of cost.*” (35–year–old, female medical officer)

### Theme 3

There were 6 subthemes found under theme 3: service delivery, infrastructure, staff turnover, staff training, changes to the immunization schedule and registration.

#### Service delivery

The respondents said that they try and take time with each parent and child so that child receives the best care and there is enough time to explain to the parents about the vaccines, the diseases that vaccines are for and possible side effects. The respondents stated that at times, the clinic is busy and nurses felt rushed to clear the crowd, and did not have adequate time to explain properly.

“*Sometimes, the clinics are full and I feel rushed, because as one child is immunized, another is waiting. But I try to take some time out and explain the vaccines and advise parents what to expect. I must, must take time out to explain. That is what I am here for.*” (31–year–old, MCHC staff nurse)

In rural settings, especially in the interiors and regional places, a staff noted that service delivery needs to improve. The nurse practitioner stated that she has worked in a rural area as well as outer islands and that immunization clinics had to be booked in advance. She said that parents had also faced issues with transport as they came by boat and depended on the tidal levels or weather conditions.

“*Having worked in rural places and regional island, children were booked in advance – especially for the MR vaccine since one vial has 10 doses in it. Some places parents can experience tide issues (low or high tide). If the fridge is not working, children will need to be booked in advance, and bring the vaccine on the same day and immunize the children. This all done bearing in mind the transport issues and the distance of the main supplier to the destination.*” (51–year–old, female Nurse Practitioner)

#### Infrastructure

Most of the health facilities were said to be lacking by the staff. The participants stated that buildings were old and needed renovation. Some of the staff stated that the waiting area were overcrowded sometimes, and parents did not have adequate seats to sit down.

“*On busy clinic days, there is crowding, and people are standing and with staff being short, we deal with angry and frustrated parents, who may have been waiting for long… standing up.*” (32–year–old, MCHC staff nurse)

#### EPI training

One of the staff at Makoi Health Centre clinic has just recently joined the MCHC. She stated that she has worked in Lautoka Hospital and now in Makoi Health Centre but has not gone through the formal EPI training. She has stated that she reads up on the immunizations so that she is able to explain to the parents, but she said that she wants to be trained in EPI since she will be working in the clinic. This sentiment was shared by most of the staff who said that they were not formally trained in EPI and that there needed to be more workshop and trainings for staff in the maternal child health clinics.

“*I have not been EPI trained yet, but I do some reading to be knowledgeable about the vaccines. I have worked in MCHC at Lautoka hospital for more than 5 years and now 2 months at Makoi. But I want to go for EPI training as I don’t want to have any problems in the future.*” (29–year–old, MCHC staff nurse)

#### Staff turnover

Some of the health centers could not get replacements for the staff that had been transferred or faced shortage. Most of the respondents felt that there was a shortage of staff and there are days when they felt overworked.

“*We need more trained staff. We get very short of staff on days that nurses go for outreach, tracing and zoning. The zone nurses also help with the immunization and it makes our work easier and clinics to finish early.*” (25–year–old, Zone staff nurse)

#### Immunization schedule

The respondents were asked about the present schedule and how they felt about it, whether there needed to any changes, additions or did not need any changes. Few of the staff (n = 14) felt that there was not a need at present to have a change in the current schedule. However, some (n = 8) felt that there was a need to have additions added to the current system.

“*I think the schedule is okay, but I think we should include meningococcal vaccine since there was an outbreak in Fiji a few years ago.*” (31–year–old, MCHC nurse)

A medical officer at Valelevu Health Centre felt that there needed to be additions to the present schedule. She stated that she has seen a few children that have presented to GOPD with severe respiratory illness or acute febrile illnesses. She also stated that there have been seen sporadic cases of meningitis and she felt that it was important to add that to the schedule.

“*I feel that it is important to have changes to the schedule. I feel like that we should include meningococcal and influenza vaccines into the schedule. I say this because we see a lot of cases of influenza in the GOPD and also a few years back, we had meningococcal outbreak.*” (35–year–old, female medical officer)

*Registration* The respondents were asked about the registration process and administration of the vaccines. They were asked how they felt about the current situation and what changes are required. At present, the information is entered into a register book at the respective health centers. Most of the staff felt that there needs to be a change in the registration process. They reported that the paper–based system was outdated and time–consuming. Some of the respondents recommended to implement electronic registration system.

“*This paper system (register book) is not ideal, as every five years we take it down so if a child is terminal or sick, there previous five years record is thrown. We are not aware of their immunization status or what their blood system or previous records were like.*” (31–year–old, MCHC staff nurse)

The other suggestion from a few of the staff was that an additional section to be added to the Patient Information System (PATIS). They said that most of the health facilities will have access to this and one can access the information on immunization status just by entering the National Health Number.

“*The Ministry can have additional option to add immunization status on the current PATIS. It is very hard to go through books and go through each record for tracing and when parents come to get their school entry card filled.*” (34–year–old, Zone nurse)

The MCHC staff also stated that they used an alternative method of updating and informing other centers of immunizing children from their centers. They said that there was a page created on FaceBook (FB) where they input the name and picture of the front page of the MCH card so that the staff at that particular center could update their records. At times, they said that they called that center and informed the staff about it.

“*There is a FB page for the EPI nurses, when other area parents come to their centers for immunization, they have to take a picture of the card that is updated and entered into their records or call the respective health center and inform them about the child receiving their immunization. Communication is the important key in the immunization process at all the centers. But this is done in keeping patient confidentiality.*” (31–year–old, MCHC staff nurse)

## Discussion

This research explored the perceptions of HCWs towards childhood immunization and immunization services in Suva, Fiji. Some of the factors identified that may be hindering the immunization services and coverage were parental factors, HCW factors and health system factors. In this study, HCWs reported that they faced some challenges in immunization services and are an important link for parents as sources of information, immunization recommendations and support.

### Barriers and challenges

Some of the barriers perceived by the HCWs were parental religious beliefs, parental knowledge, attitudes and parental hesitancy. Understanding these barriers and motivators behind parents’ decisions for immunization provided valuable insights that has potential to shape vaccine messaging, recommendations and policies [33]. While parental attitude can be a barrier, it can also act as an enabler.

Other studies have shown that social or physical factors (vaccine availability, finances, cost of transportation and childcare, work conflicts, family dynamics) and health care factors (access to health services, immunization policies, hours of clinic operation, shortage of personnel, waiting time, missed opportunities, health care professionals’ attitudes and practices) could be some of the barriers to immunization [34, 35].

HCWs play an important role in immunization as they are the source of information and motivation for parents/ caregivers. The respondents in this study stated that they would always reassure parents and listen to their concerns and try to alleviate any concerns that the parents may have as parents put their trust on HCWs and that correct information imparted and have positive attitudes towards immunization and had a high confidence in the advice given by the HCWs [36] and are cited by parents as the most frequent sources of information regarding immunization [37].

HCWs answering questions and concerns would enhance trust towards the provider and compliance with immunization and that providers should not have a dismissive or authoritative attitude and let parents initiate the topic of vaccines and concerns [38]. Respect for HCWs and their profession influences the trust [39] and the attitudes of HCWs towards the parents are important motivators for parent in taking advantage of the immunization services [40]. Studies have shown that for parents’ trust was more important than individual attitudes regarding vaccine behavior and also as vaccine decision–making is a process and not a snap decision, therefore it is important for a provider to establish trust and develop rapport as way of balancing power between the patient and provider and allow the patient to lead the vaccine discussion [38].

#### HCWs knowledge and attitude

Knowledge and effective immunization communication has the potential to improve the HCWs’ ability to increase parental acceptance of childhood immunizations. The knowledge of effective immunization communication has the potential to improve the HCW’s ability to increase parental acceptance of childhood immunizations [41].

HCWs have obligations to work for the betterment of their clients, being trustworthy, have a high level of clinical and judgement skills and fulfilling legal obligations [39].Parents believe in the protective effect of vaccines and follow the advice of their children’s health care which indicated the critical opportunity for doctors and HCWs to respond to parental concerns about vaccine safety [42], with vaccine safety noted to be the main concern for most parents [43]. Immunization providers and other HCWs have an important role to play in the decision–making process of parents by providing complete information on vaccines, especially the risks and benefits [44].

There is a lot of misinformation readily available on social media and in social groups, and it will be easy for parents to form misconceptions and distrust regarding vaccines and immunization, HCWs and the health system. The trustworthiness of the information source (pharmaceutical industry, government, HCW, or community member) impacts the credibility of the information [43]. The HCWs can establish trust by displaying empathy and concern when imparting information on vaccines, vaccine safety and the experience of HCWs increases the relationship and trust [38].

There needs to be effective strategies developed to confront vaccine hesitancy and reliable and trustworthy sources available for parents to access information regarding immunization and vaccines [43]. HCWs should further strengthen their interpersonal and communication skills [39] and given the pivotal role that HCWs play in parental immunization decision–making, further investigations into how providers communicate with vaccine hesitant parents is the important first step in determining which communication practices are effective [41].

All participants were noted to have positive attitudes and perceptions towards immunization and stated that they have immunized their children according to the present schedule and would get their child immunized if any new vaccines are introduced. Parents need reassurance at each visit and get concerned when the child cries when receiving the injectable forms of the vaccine. The participants stated that parents need to be informed that this is normal and parents are made aware of the possible side–effects of fever, swelling at the injection site and HCWs need to remind parent of the next appointment. Missed opportunities to administer all the scheduled vaccines at the same visit indicate low or no vaccine stocks, mistakes in identifying which vaccines are due, reluctance to vaccinate a sick child or to administer multiple vaccines at the same visit [17].

In our study, the participants acknowledged that vaccines were important, and they always attempted to build trust and to reassure their clients. Whenever they encountered a hesitant parent or defaulters, they reported that they always tried to talk to the parents and find the reasons and tried to alleviate the parental concerns. To maintain a successful vaccination program, it is important to identify the HCWs who are vaccine–hesitant to understand the causes of their hesitancies and to develop tailored strategies to address this [45]. The public health system can serve to educate the community about vaccines and VPDs and reassure them that the vaccines are safe [43].

#### Improving the services and challenges

Some of the participants reported that they had not had any formal EPI training and obtained their knowledge on their own. The quality of immunization can be strengthened by providing training and support to immunization service providers to reduce the missed opportunities for vaccination and improve the quality and safety [46]. Most of them felt that there needs to be regular training programs so that the staff are knowledgeable and able to improve the quality of services at their facilities. Some of the participants felt that at times the work environment affected their service output and at times they do not have time to attend to each parent and their concerns.

A few of the HCWs felt that the current Fiji Immunization Schedule would benefit with the addition of influenza and meningococcal vaccines. A few of the GOPD staff had encountered sick children presenting to them in the GOPD and the case numbers has increased over the years. Access to health facilities acts as a barrier for parents in regional areas and parents utilize a boat or carrier to get their child to the clinics or depended on the tidal times.

Electronic immunization registers can be invaluable for improving and measuring coverage and data could be extracted easily – the functions could include identifying children due for immunizations, sending reminders which improves the timeliness of the immunization, improve the supply chain and enable HCWs to track children across multiple facilities, thus improving vaccine uptake [47]. Equitable access to immunization to achieve high coverage can be enhanced through financial and technical support for program strengthening and vaccine introductions, community engagement to increase vaccination acceptance and demand, collection of vaccination data and improving the services [48].

In Fiji, Suva had a lockdown from April 3, 2020 to April 17, 2020 when the first wave of COVID–19 hit Fiji [49]. The second wave of the COVID–19 outbreak caused another lockdown in the Suva–Nausori corridor and the Nadi–Lautoka corridor with movement being completely restricted in Suva from May 14, 2021 to May 19, 2021. During this time all the MCHCs were closed, and this caused a lot of children to miss out on their scheduled vaccines. COVID–19 pandemic is a cause of concern to routine immunizations and stated that children had missed their RIs during the COVID–19 lockdown [50]. According to the official data from WHO and UNICEF, 23 million children have missed out on basic childhood vaccines through routine health services in 2020, the highest number since 2009 and 3.7 million more than in 2019 [14]. Pools of unimmunized children can expand further in lockdowns, leaving them susceptible to VPDs and suggested for tailored interventions to promote immunization and safe service delivery during the lockdowns [50].

## Conclusion

This study showed that the health workers were confident to attend to any queries and issues parents may have regarding the immunization and vaccines, regardless of the department they worked in at the health facility. The study also found that staff reported some difficulty in defaulter tracing as the registered addresses are not correct or the family had moved, therefore could not be classified as true defaulters. The study highlighted that GOPD health workers encountered parents who hadmissed or not taken all the vaccines and these children present with illnesses such as severe respiratory illnesses, measles, or sepsis. The study found that communication and empathy is essential to get a good parent–HCW relationship and parents responded well to staff that were approachable. The study highlighted that workers need to be tactful in how they handle vaccine hesitant parents and that they should counsel and advise parents to alleviate their fears. Despite the success of immunization in Fiji, there still needs to be improvement in the immunization services in Fiji in terms of training, infrastructure and staffing. It was reported that staffing was short and most staff had to multi–task in the MCHCs.

More research is needed on how to utilize social media to raise awareness and influence parents in their decision–making process regarding immunization. A greater understanding of this can maximize the efficacy of immunization strategies to increase coverage. Further studies are needed nationally to determine the perceptions of HCWs towards immunization and how the services can be improved.

### Limitations

Findings of this study must be interpreted looking at the context of the study limitations., The research is limited to three s health centers in Suva and the findings cannot be generalized to the other HCWs in other centers. Another limitation was that the study was conducted in only three health centers in Suva, hence leaving out other health centers in rural and remote areas, where the workers might have different perceptions and experiences. Furthermore, the study only included primary HCWs, and it would be ideal to include other health practitioners such as private general practitioners and health workers in major hospitals.

## Electronic supplementary material

Below is the link to the electronic supplementary material.

Supplementary Material 1

## Data Availability

The datasets generated and/or analyzed during the current study are not publicly available due ethical restrictions but are available from the corresponding author on reasonable request.
