# Supplementary material for: Perceptions of Healthcare Workers (HCWs) towards childhood immunization and immunization services in Fiji: a qualitative study
Source: BMC Pediatr. 2022 Oct 21;22:610. doi: 10.1186/s12887-022-03665-9 (PMC9585826; doi:10.1186/s12887-022-03665-9)
Supplement: Supplementary file 1 — Supplementary Material 1 [file 12887_2022_3665_MOESM1_ESM.docx]

**Semi-structured Questionnaire for HCWs**

**Section 1: Demographic Questions**

1. What is your age?

2. What is your marital status?

3. What gender do you identify with?

4. What is your ethnicity?

5. Where do you live?

**Section 2: Focus group discussions**

1. What is your designation at the health centre?

2. How many years of service have you had?

3. How long have you worked at the health centre? Or how long have you been in the Maternal Child Health Clinic?

5. Have you had training for the Expanded Programme on Immunisation for Fiji? Do you know the Expanded Programme on Immunisation for Fiji?

6. Do you have knowledge about the vaccines on the Fiji Immunisation Schedule?

7. How do you feel about the vaccines and the Fiji Immunisation Schedule?

8. Do you have any children, and would you consider getting your child immunised? If you do not have any children presently, would you get your child immunised?

9. Do you think the coverage is enough?

10. How confident are you in answering any questions any parent may have regarding the vaccines?

11. Do you feel that there needs to be any more additions to the schedule? Why?

12. How do you feel about the cooperation from the parents/ caregivers attending the clinic?

13. What are the challenges that you face in the clinics?

14. Do you have time to explain to the parents the effectiveness of the vaccines?
